# Network Modeling of MDM2 Inhibitor-Oxaliplatin Combination Reveals Biological Synergy in wt-p53 solid tumors

**DOI:** 10.18632/oncotarget.269

**Published:** 2011-05-16

**Authors:** Asfar S. Azmi, Sanjeev Banerjee, Shadan Ali, Zhiwei Wang, Bin Bao, Frances W.J. Beck, Main Maitah, Minsig Choi, Tony F. Shields, Philip A. Philip, Fazlul H. Sarkar, Ramzi M. Mohammad

**Affiliations:** ^1^Department of Pathology, Wayne State University School of Medicine; ^2^Departments of Oncology, Karmanos Cancer Institute, Wayne State University School of Medicine, Detroit, Michigan

**Keywords:** MDM2 and p53, MI-219, oxaliplatin Network Modeling

## Abstract

Earlier we had shown that the MDM2 inhibitor (MI-219) belonging to the spiro-oxindole family can synergistically enhance the efficacy of platinum chemotherapeutics leading to 50% tumor free survival in a genetically complex pancreatic ductal adenocarcinoma (PDAC) xenograft model. In this report, we have taken a systems and network modeling approach in order to understand central mechanisms behind MI219-oxaliplatin synergy with validation in PDAC, colon and breast cancer cell lines. Microarray profiling of drug treatments (MI-219, oxaliplatin or their combination) in capan-2 cells reveal a similar unique set of gene alterations that is duplicated in other solid tumor cells. As single agent, MI-219 or oxaliplatin induced alterations in 48 and 761 genes respectively. The combination treatment resulted in 767 gene alterations with emergence of 286 synergy unique genes. Ingenuity network modeling of combination and synergy unique genes showed the crucial role of five key local networks CREB, CARF, EGR1, NF-kB and E Cadherin. The network signatures were validated at the protein level in all three cell lines. Individually silencing central nodes in these five hubs resulted in abrogation of MI-219-oxaliplatin activity confirming their critical role in aiding p53 mediated apoptotic response. We anticipate that our MI219-oxaliplatin network blueprints can be clinically translated in the rationale design and application of this unique therapeutic combination in a genetically pre-defined subset of patients.

## INTRODUCTION

Network modeling and systems biology are important tools that are finding applications in the area of drug discovery [1]. This technology allows real-time simulation of how biological molecules function in coordination to achieve a particular outcome, consequently providing tremendous power of predicting the drug response in terms of the effect of modulating the function of a given molecule or pathway [2]. A network perspective of drug targets has direct implications in drug discovery process since it changes the target entity from a single molecule to entire molecular pathways or cellular networks. Such technologies are crucial for identifying and understanding the mechanisms of potential target candidates in complex diseases where core de-regulatory networks are still being identified [3].

Biological interaction networks have been available to the scientific community for more than a decade, but only in the last few years has the concept of network biology found its application in the field of cancer drug discovery. Despite its shortcomings, the initial version of human interactome networks are now of sufficient quality to provide clinically useful information [4,5]. Such integrated analyses may lead to the identification of pathways and help in our understanding of single drug mechanism of action, synergy between two drugs, or enhance our knowledge as to how one drug modulates the effect of another given drug. Thus far, network analysis has facilitated the prediction of possible molecules affected by specified perturbations of up and downstream targets by different drugs. Such predictions can be applied to the development of clinically relevant drug combinations. This is important for understanding drugs that are designed against master regulators such as p53, known to regulate a variety of targets and is the focus of this study.

p53 (often considered guardian of genome) [6] is found mutated in about 50% of all cancers [7,8]. In the remaining 50%, p53 is wild type (wt-p53), however, its function is inhibited mainly by the cellular oncoprotein MDM2 [9,10]. Therefore, wt-p53 reactivation by blocking MDM2-p53 interaction using small molecule inhibitors is considered an effective therapeutic strategy for the treatment of wt-p53 cancer [11-14]. Over the last decade, many groups including ours have extensively worked on developing small molecule inhibitors of MDM2 (here MI-219, developed in collaboration with Ascenta Therapeutics [15]) and tested them against multiple cancers including lymphoma [16], PDAC, colon and breast [17,18]. Our laboratory has also investigated novel and potent combinations of such MDM2 inhibitors with standard chemotherapy and demonstrated synergy with platinum drug treatments (but not gemcitabine) that resulted is tumor free survival in PDAC xenograft models [19]. Such strong preclinical evidence has accelerated the development of MDM2 inhibitors towards clinical application [20].

We have found that MI-219 when combined with oxaliplatin can induce superior growth inhibition in wt-p53 PDAC [21]. This synergistic efficacy was not restricted to a PDAC tumor models and could be translated to other wt-p53 solid tumors. Although investigations from our laboratory certainly prove the potential of these inhibitors against wt-p53 tumors, still, our knowledge of the mechanism of action of these inhibitors, especially their combination synergy with platinum drugs, is incomplete. This is because MDM2 has protein partners both upstream and downstream but yet are independent of p53 [22] and we are still learning the intricacies of the p53-MDM2 pathway, its role in tumorigenesis and the influence of additional regulatory networks on these two multifaceted proteins [23,24]. Recently, we have proposed that decoding the complexity of targets associated to both p53 and MDM2 requires a network centric approach considering global interacting proteins partners without loosing key details [21, 25]. Such holistic approach will help in better understanding of MDM2 inhibitor mechanism of action either alone or in combination with various chemotherapeutic drugs.

In this paper, we utilized a systems biology and network modeling approach to delineated the underlying mechanisms governing the synergy between MI-219-oxaliplatin in PDAC cell line. We proposed and provided data to demonstrate that our synergy networks signatures could be utilized for rationally designing clinical trials of MI-219-oxaliplatin in a genetically pre-defined subset of patients with not only PDAC, but also in patients with other types of cancer, providing an understanding for a more optimal outcome.

## RESULTS

### MI-219 and oxaliplatin is a highly potent combination in wt-p53 solid tumors

**Figure 1:**
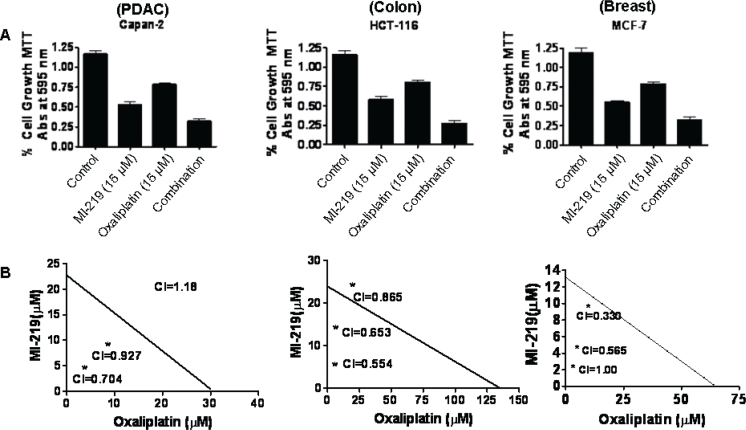
MI-219 synergizes with Oxaliplatin leading to enhanced growth inhibition in wt-p53 cancer cells Figure 1 A) Three wt-p53 cancer cell lines Capan-2 representing PDAC; HCT116, colon cancer and MCF-7, a breast cancer cell line were seeded at a density of 3000 cells per well in 96 well plates. After 24 hrs the media was aspirated and cells were exposed to either MI-219 (0-30 μM) for Capan-2 or (0-15 μM for MCF-7 and HCT-116); oxaliplatin (0-30 μM) for Capan-2 or (0-15 μM for MCF-7 and HCT-116) or combination of MI-219 and Oxaliplatin (multiple concentrations in the ratio of 1:1 for isobologram analysis) for 32 hrs. At the end of the treatment period, 20 μL of MTT (Sigma St Louis USA) solution (5 mg/ml PBS) was added to each well and incubated at 37 °C for 2 hrs. After the incubation period was over, the media was aspirated and re-fed with 20 μL of DMSO per well following rapid mixing on a plate shaker. After 15 minutes of shaking the color developed was read at 595 nm using a ELISA Plate reader (TECAN Durham USA). Figure 1B) Isobologram analysis of MI-219-oxaliplatin combination treatment using CalcuSyn software a (CI<1 indicates synergy) and is observed in all cell lines.

**Figure 2:**
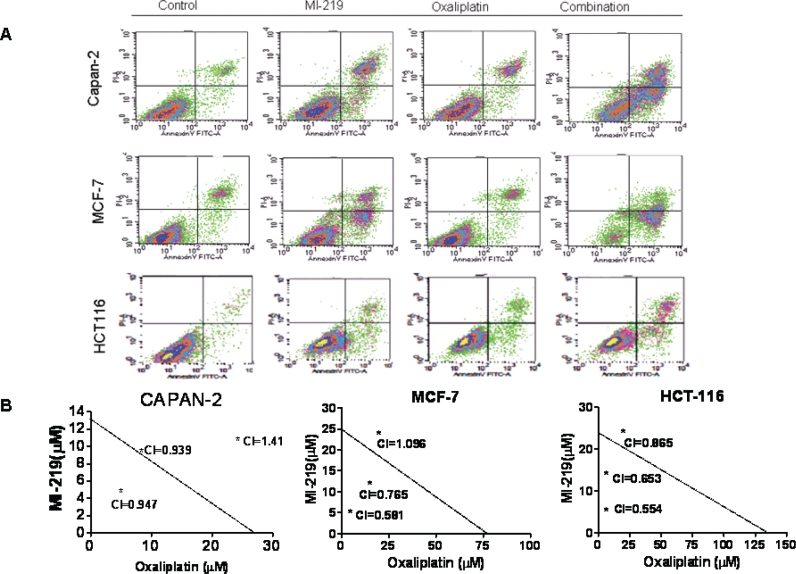
MI-219-oxaliplatin induces synergistically enhanced apoptosis [Upper Panel] Capan-2, MCF-7 and HCT-116 cells were grown in six well plates and treated with either MI-219 (15 μM) or oxaliplatin (15 μM) or the combination of MI-219 and Oxaliplatin (15 μM each) for 32 hrs. At the end of the treatment period, cells were trypsinized washed twice with PBS. The pelleted cells were tested for apoptosis using Annexin V FITC apoptosis assay according to the manufacturers guidelines (Biovision CA, USA Cat # K-101-100). [Lower Panel] Isobologram analysis of MI-219-oxaliplatin indicates synergy.

**Figure 3:**
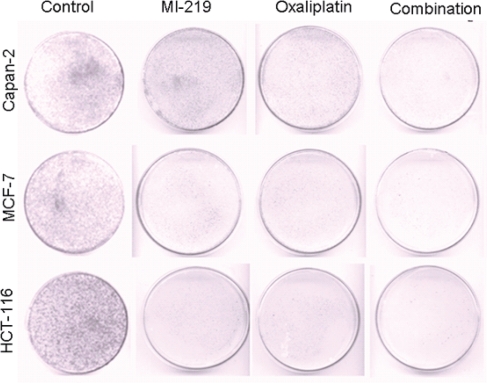
MI-219-Oxaliplatin suppresses colonogenic potential in different cancer cells Cells were treated as described in Figure 1 and after the incubation was over, cells were plated in 100 mm petri plates in a density of 500-1000 cells depending on colonogenic potential. The plates were incubated in low CO_2_ days at 37°C in a 5% CO_2_/5% O_2_/90% N_2_ incubator. The colonies were stained with 2% crystal violet and counted. The surviving fraction was normalized to untreated control cells with respect to clonogenic efficiency.

**Figure 4:**
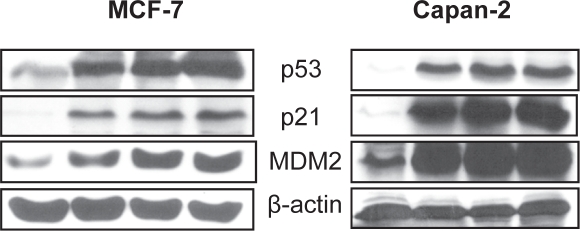
MI-219-Oxaliplatin super induces p53 compared to single agent treatment MCF-7 and Capan-2 cells grown in 100 mm petri plates were exposed to either (i) [Left to Right Lanes] vehicle control; (ii) MI-219 15 μM; (iii) Oxaliplatin 15 μM and (iv) combination of MI-219 and Oxaliplatin (15 μM each) for 32 hrs and protein was isolated as described in Methods section. Equal amounts of protein were analyzed for p53, MDM2 and p21 by Western Blotting. The membranes were re-probed with β-actin as loading control. Note in both cell lines the combination treatment lead to enhanced expression of p53, p21 and MDM2 in both cell lines.

In order to understand mechanism of action of MI-219-oxaliplatin efficacy we first performed growth inhibition and apoptosis studies in wt-p53 Capan-2, HCT-116 and MCF-7 cancer cells. As can be seen from results of Figure 1A, MI-219-oxaliplatin treatment resulted in synergistically enhanced growth inhibition in all three types of tumors. Isobologram analysis of the combination treatment showed statistically significant synergy (Combination Index CI<1 in all three cell lines Figure 1 B). Then, we performed apoptosis assays that showed similar trend with maximal apoptotic death observed in MI-219-oxaliplatin treatment group (Figure 2). This combination was also found to be synergistic. We also evaluated the inhibitory effect of colony formation in all the three cell lines. In concordance with our growth inhibition and apoptosis results, MI-219-oxaliplatin blocked clonogenic capacity of the tested cell lines (Figure 3). The above multiple assays clearly demonstrate a synergistic interaction between the two drugs that leads to enhanced growth inhibition in three different solid tumor cell lines. We further evaluated the consequence of combination treatment on activation of p53 pathway using western blot analysis. As can be seen from results of Figure 4, compared to single agent the combination treatment resulted in slightly superior p53 and p21 re-activation. However, in order to prove that this is indeed a biological synergy, we performed network modeling on microarray obtained from individual and combination treatment in the same cells.

### Microarray profile MI-219-oxaliplatin indicates modulation of unique set of genes

**Figure 5:**
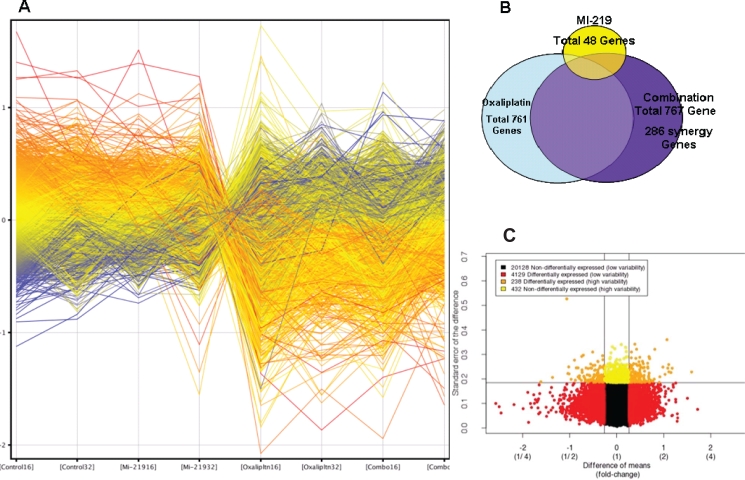
Microarray profiling of MI-219-Oxaliplatin treatment in Capan-2 PDAC cells [A] Human Illumina HT-12 microarrays were performed on micro array grade RNA isolated from Capan-2 cells treated with either DMSO; MI-219 (15 μM); oxaliplatin (15 μM) or their combination for 16 and 32 hrs. Note: each treatment and time point has a unique set of gene expression profile (here color coded). All results represent at least triplicate samples except for oxaliplatin 32 hrs that was run in duplicate. [B] Biological Venn diagram showing each treatment induces unique set of gene changes (48 for MI-219 single agent treatment; 727 for oxaliplatin treatment; 767 for combination treatment and 286 genes as synergy unique genes. [C] Test for differential expression and variability indicates biomarker of response. Dataset of 24927 genes was analyzed in combination 32 hr vs. control 32 hrs. Differential gene expression: Combo vs Control (p = 0.01) Red = differentially expressed low variability Orange = differentially expressed high variability (potential biomarker of response). All values represent triplicate measurements with p value 0.01.

Systems and network modeling considers proteins as components of a highly interactive network that cross-talks with different partners in its vicinity. This technology can also be used to understand drug target response networks considering each perturbation in its entirety without loosing key detail. Therefore, in order to delineate the mechanism of synergy between MI-219-oxaliplatin, microarrays were performed on RNA isolated from single agent or combination treatment. As can be seen from results of Figure 5A, each treatment (control, MI-219, oxaliplatin and combination color coded) has a unique set of associated gene signature. As expected of a targeted drug, MI-219 as single agent induced a minimal of 48 gene alterations (Figure 5B smallest circle). Oxaliplatin, a cytotoxic agent that has a well recognized toxicity, altered 761 genes. The combination treatment resulted in a total of 767 gene changes (summarized in supplementary Table 1). However; of paramount significance is the emergence of 286 synergy-unique genes that are not found in either single agent treatment. This sizable number of genes restricted to the combination treatment is indicative of a true biological synergy between MI-219 and oxaliplatin. Furthermore, our analysis shows that the entire set of genes (24,927) could be pooled into four different groups (20,128 non-differentially expressed with low variability; 4,129 differentially expressed wit low variability; 238 differentially expressed with high variability and 432 non-differentially expressed with high variability) (Figure 5C). Such a large number of differentially expressed genes with high variability are indicative of biomarker of drug response.

**Figure 6:**
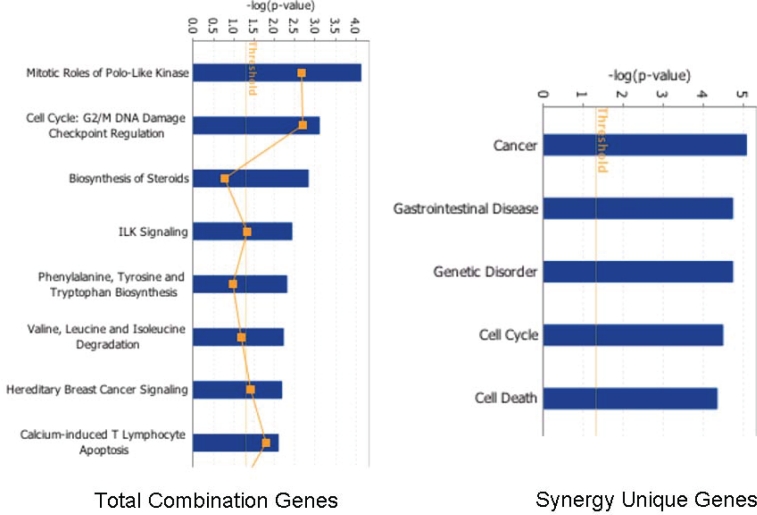
Combination synergy represents biologically meaningful process: Analysis of biologically activated functional pathways in combination treatment in (left panel) total combination genes vs synergy unique genes (right panel). Note: enrichment of canonical functional pathways in combination datasets vs. restricted set of pathways in synergy unique datasets. Cutoff range was set at 1.7 in the ingenuity pathway analysis systems.

Meta analysis of the total combination genes using Ingenuity Pathway Analysis (IPA) software showed statistical enrichment in canonical pathways (pathways include mitotic roles of polo-like kinases, cell cycle: G2/M DNA damage checkpoint regulation, steroid biosynthesis, ILK signaling, phenylalanine, tyrosine and tryptophan biosynthesis pathways, valine, leucine and isoleucine degradation, hereditary breast cancer signaling and calcium induced T-lymphocytes apoptosis (Figure 6). However, similar analysis of the synergy- unique genes showed an entirely different set of statistical pathway enrichment. In this case, the restricted set of pathways was for cancer, gastrointestinal disorder, genetic disorder, cell cycle and cell death (Figure 6). These results are a further validation of the fact the combination synergy represents biologically meaningful pathways and not resultant from random set of events.

### Network modeling of MI-219 mediated changes at different time points

In order to understand the mechanism of MI-219 induced changes on oxaliplatin treatment, we performed ingenuity modeling of the microarray datasets at different time points. As can be seen from results of Figure 7A, the initial response to MI-219 treatment (at 16 hrs) is the activation of cell survival genes through NF-κB complex formation. However, at later time points (32 hrs), this survival signaling is diminished and we observe emergence of multiple de-regulated signaling gene networks that are indicative of cell death (Figure 7B). In order to verify whether, these genetic events could be captured at the functional level (protein expression), we performed western blot analysis on cellular lysates obtained at the two treatment time points. Figure 7C shows that at 16 hrs p65 (component of NF-κB) is highly expressed while secondary hub such as EGR1 is not. However, at 32 hrs, p65 expression is diminished while EGR1 is activated. Of significance is the fact that the protein expression pattern induced by MI-219 in PDAC (Capan-2 cells) could be replicated in an entirely distinct tumor cell line HCT-116. Once we obtained insight of the mechanism/perturbations induced by MI-219 as a single agent, we explored that changes in combination treatment and are discussed in the forthcoming passages.

**Figure 7:**
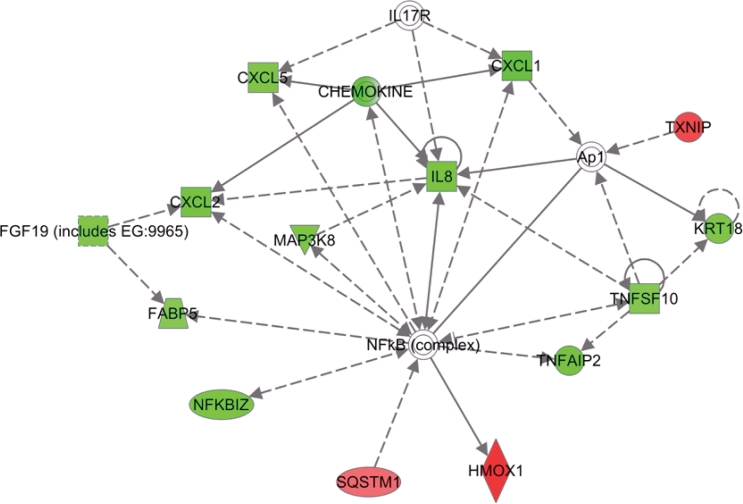

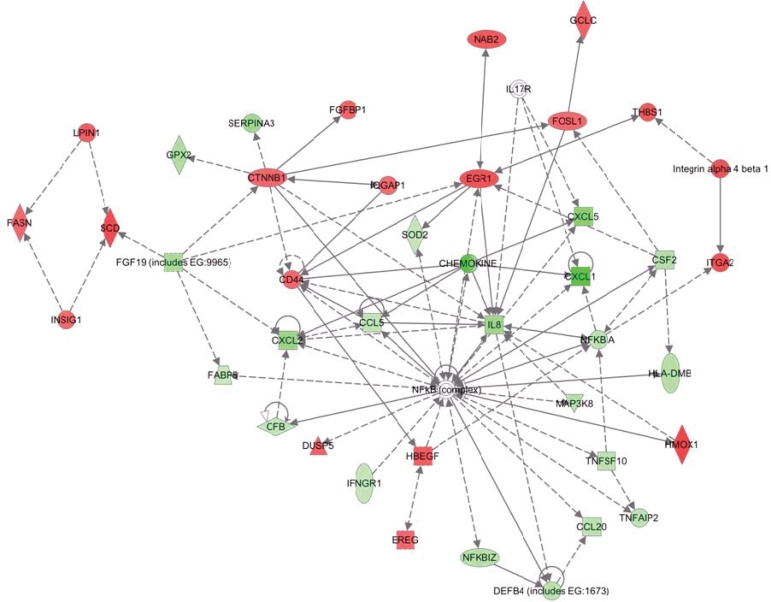
Network modeling and validation of MI-219 single agent treatment at different time points [A] Ingenuity pathway analysis of primary events mediated by MI-219 single agent treatment at 16 hrs showing activation of NF-κB central hub. [B] Analysis of MI-219 single agent treatment mediated secondary events at 32 hrs showing de-regulated signaling and activation of secondary hub EGR1. Analysis presented in A and B are that of three biological replicates…

**Figure 7C:**
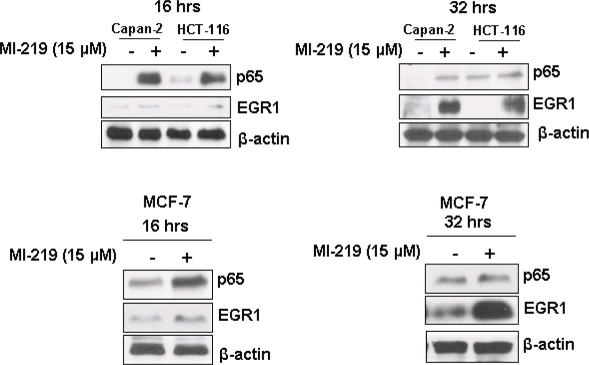
Network modeling and validation of MI-219 single agent treatment at different time points [C] Validation of primary and secondary gene changes at the functional level i.e. protein expression. Note activation of NF-κB p65 (p65) at 16 hrs and no significant changes in EGR1. However, at 32 hrs, p65 expression is lost and EGR1 hub is activated in all three cell lines. β-actin was used as loading control. Blots are representative of three independent experiments.

### Change in information flow upon combination treatment: Towards cell death

**Figure 8:**
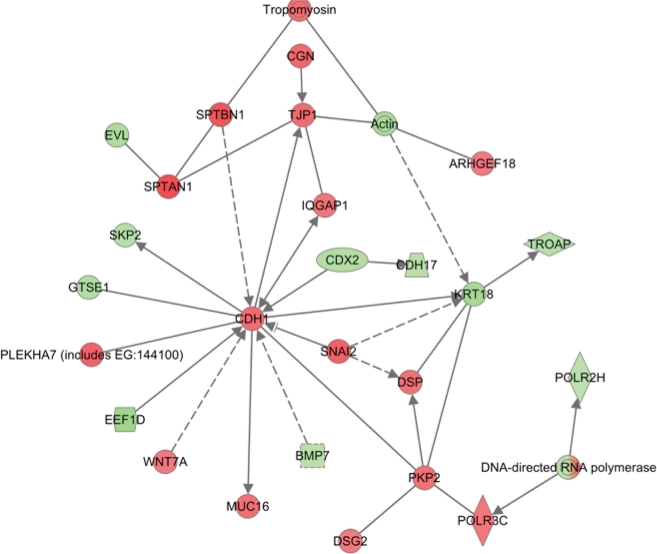
Ingenuity network analysis of total combination genes showing change in information flow in cells undergoing apoptosis: E-Cadherin represented by CDH1 anti-tumor module is activated in combination treatment Red is indicative of genes going up and green depicts genes suppressed. All analysis presented are means of three biological replicates.

**Figure 9:**
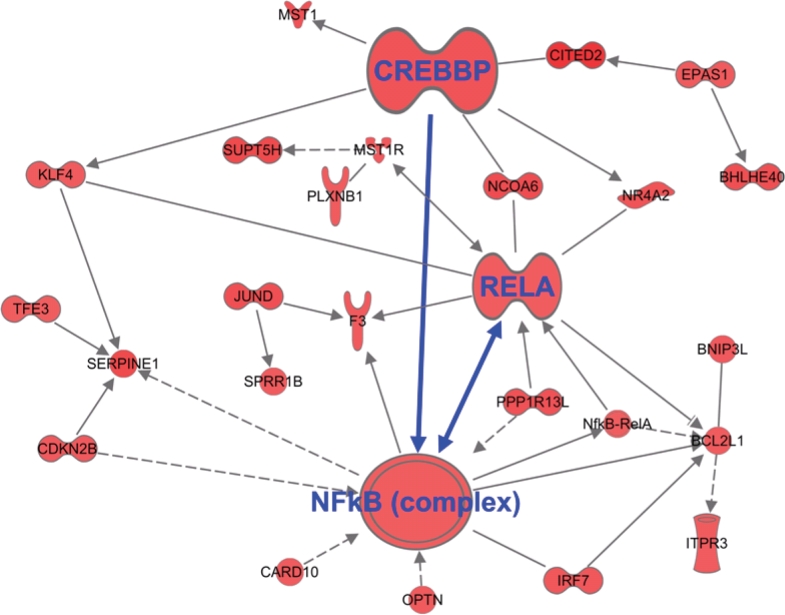
Global changes in gene homeostasis showing activation of NF-kB-CREB axis in synergy unique dataset Triplicate datasets of the synergy unique genes were analyzed using ingenuity pathway analysis software and demonstrated activation of NF-kB module in tandem with CREB. Note all genes shown here are up-regulated.

Apoptotic cells undergo a rapid change in genetic information directed towards de-regulated signaling leading to cell death. To provide evidence for this, our analysis of the combination genes at late time point (32 hrs) showed dramatic changes in information flow that led to the activation of the anti-tumor E-Cadherin hub, (CDH1 protein) (Figure 8; Red showing up regulated genes and green showing down regulated genes). This is in agreement with earlier studies showing that in addition to regulating cell junction, E-Cadherin network responds to different stresses leading to apoptotic cell death [26]. The second activated hub observed in the 32 hr treatment group was the NF-κB complex-CREB axis (Figure 9). This was a different expression pattern compared to MI-219 single agent treatment that showed suppression of NF-kB. These data further provide evidence for the uniqueness of each treatment signature. Our analysis also showed the activation of EGR1 network (Supplementary Figure 1) and p53-CDKN2A hub (Supplementary Figure 2) that in principle drives optimal p53 activation. The activation of multiple hubs driving p53 is indicative of a true synergy between MI-219-oxaliplatin and suggests clinical testing of this combination would be of benefit to patients with wt-p53 solid tumors.

### Validation of synergy genes at the functional level

**Figure 10:**
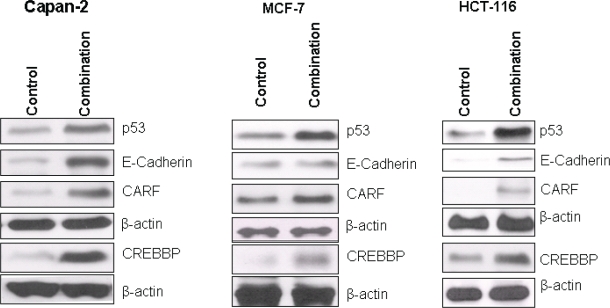
Validation of network genes at the protein level: Capan-2, MCF-7 and HCT-116 cells were seeded in 100 mm petri dishes at the density of 1 million per plate in 10 ml media Once the cells achieved 60-70% confluence, media was aspirated and replenished with fresh media containing either vehicle alone of MI-219+Oxaliplatin combination (15μM each). The cells were incubated for 32 hrs followed and at the end of the treatment period, cells were harvested, washed twice in PBS followed by extraction of protein for western blot analysis. Preparation of cellular lysates, protein concentration determination and SDS-PAGE analysis has been previously described (28). Equal amounts of protein were analyzed for p53, E-Cadherin, CARF and CREBBP by Western Blotting. The membranes were re-probed with β-actin as loading control. Note that the patterns of gene expression were similar in all three cell lines.

The changes in gene expression observed in the microarray analysis of MI-219-oxaliplatin combination synergy provide inconclusive evidence of the mechanism of action unless their functional/effector translational product is validated at the protein level. Therefore, in order to validate our network findings at the functional (or biological) level, we analyzed the protein product of prominent hub genes observed in the synergy using western blot analysis. The protein expression analysis was performed in PDAC, colon and breast cell lines in order to assess whether the uniqueness of the synergy genes could be translated to other tumors as well. As can be seen from results of Fig 10, along with the enhancement of p53, combination treatment enhances CREBBP, E-Cadherin and CARF protein expression in all the cell lines tested. That this was observed in two tumor cells lines distinct from PDAC clearly confirms that the observed activity of MI-219 and oxaliplatin is not a cell-specific phenomenon. These results are of paramount significance since they indicate that MI-219-oxaliplatin combination can become an effective for a wide range of wt-p53 tumors and not restricted to PDAC.

### siRNA silencing confirms the crucial requirement of synergy unique genes in MI-219-Oxaliplatin combination efficacy

**Figure 11:**
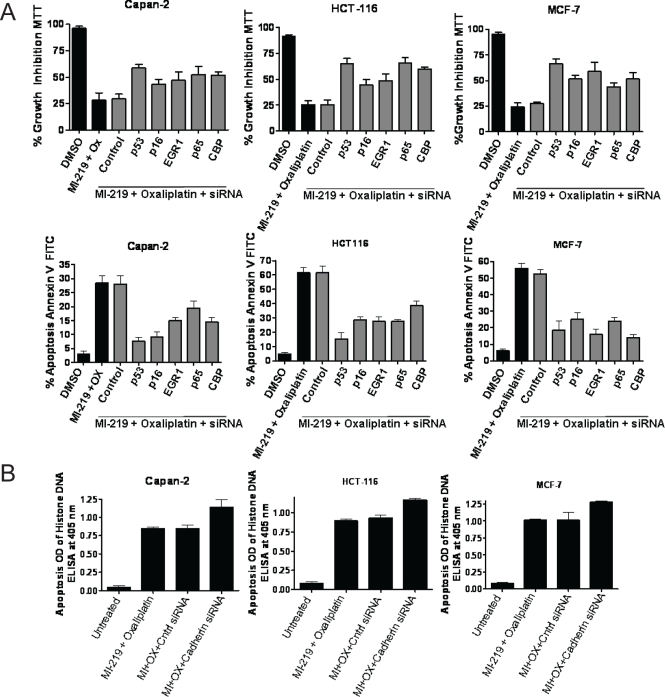
siRNA silencing of key hub genes abrogates MI-219-oxaliplatin efficacy [A] Capan-2, MCF-7 and HCT-116 cells were grown in 96 well plates (for MTT Top Panel) and 6 well plates (for Annexin V FITC apoptosis Lower Panel). Semi confluent cells were treated with either DMSO; control siRNA or p53/CBP/EGR1/p16/p65 siRNAs for 5 hrs in serum free pencillin streptomycin free media for 5 hrs. After incubation, siRNA containing media was aspirated and cells were allowed to grow overnight in normal media. Following the overnight incubation, cells were exposed to MI-219-oxaliplatin combination for 32 hrs. Annexin V FITC apoptosis analysis was performed according to the manufacturer's guidelines (Biovision CA, USA Cat # K-101-100). [B] siRNA silencing of Cadherin under similar experimental conditions. All values [in A and B] represent Mean + S.D of triplicate experiment

Once we establish the enhancement of CBP300 (a CREBBP), CARF, E-Cadherin and NF-κB-p65 (p65) at the protein level, we sought to investigate the effect of silencing individual genes and validate their role in the observed synergy between MI-219-oxaliplatin. We individually knocked down the above mentioned genes and tested the Capan-2, HCT-116 and MCF-7 cells for growth inhibition and apoptosis by MI-219-oxaliplatin. First, we analyzed the role of p53 in the observed apoptosis by MI-219 and oxaliplatin. As can be seen from results of Fig 11, siRNA knockdown of p53 abrogated MI-219-oxaliplatin mediated growth inhibition (MTT Top Panel) and apoptosis (Annexin V FITC Bottom Panel) in all the three cell lines. These results proved that p53 is central player in the efficacy of MI-219-oxaliplatin. Following this, we individually knocked down CREBBP, EGR1, CDKN2A (p16) and p65 and found that siRNA silencing suppresses the efficacy of MI-219-Oxaliplatin in three different cell types (data not shown). Most interestingly, silencing E-Cadherin exhibited the opposite effect and enhanced the potential of MI-219-oxaliplatin (Figure 12). These results support our previous ingenuity gene networks studies (Figure 8) where we found changes in information flow in the E-Cadherin hub in response to combination treatment.

## DISCUSSION

In this clinically translatable study, we have, for the first time, used integrated network modeling and systems biology to delineate the mechanism of synergy between MDM2 targeted small molecule drug (MI-219) and oxaliplatin in wt-p53 solid tumors. Based on these novel results, we anticipate that our presented information will aid in the clinical design of this potent combination in a genetically predefined subset of wt-p53 carrying cancer patients and will be a step towards tailored therapy.

Our laboratory has been focused on delineating the molecular mechanisms of action of a potent MDM2 inhibitor MI-219 in solid tumors. MI-219 is a specific, orally active, low molecular weight inhibitor, that binds to the p53 binding pocket of MDM2 and disrupts the MDM2-p53 interaction leading to apoptosis via reactivating wt-p53 in wt-p53 cancer cells [27,28]. However, this inhibitor is, by itself, a non genotoxic agent. We are of the opinion that for effective and clinically beneficial activation of p53, it is logical to combine an MDM2 inhibitor with genotoxic drugs that activate p53. Indeed, MDM2 inhibitor synergizes with platinum drugs leading to 50% tumor free survival in PDAC xenograft model [19]. Earlier work by Blagosklonny and group have demonstrated that a combination of MDM2 inhibitors and DNA-damaging drugs super-induce p53 and that was considered as a mechanism of synergy [29]. However, the precise mechanism of this synergy has not been fully characterized at the molecular level. Additionally, a number of critical networks including microRNA circuits have been shown to crosstalk with p53 family members p63 and p73 that ultimately governs therapeutic efficacy of drug treatment [30]. Given the complex network of interaction between MDM2 and p53 that ultimately governs apoptosis, it is reasonable to predict that analyzing the local network of crucial members may help understand and elucidate the cellular response to MI-219. In the case of MI-219, elucidating important key protein/pathways will help us in identifying patients who are more likely to respond to MI-219 treatment, which will provide molecular guidance for conducting rationally designed and tailored combination trials.

In this report, we demonstrated that MI219-oxaliplatin combination has far superior anticancer effects than either agent alone in three wt-p53 yet distinct solid tumors types (Pancreatic, Colon and Breast). Isobologram analysis of the combination ratios confirmed that MI-219 and oxaliplatin work in a synergistic (CI<1 in all cases) and not in an additive manner. However, lack of mechanistic studies supporting this synergistic combination in a wide spectrum of tumors suggests that further research is necessary. Taking a systems biology approach, we investigated whether the observed synergy between MI-219 and oxaliplatin is indeed the result of orchestrated network of molecular events that drive p53 reactivation towards enhanced apoptosis. As expected of a targeted agent, global analysis of genes from microarray profiling showed that MI-219 treatment resulted limited set of only 48 gene alterations. On the other hand, the well recognized cytotoxic agent oxaliplatin induced alterations in a larger number of genes (761 genes). The combination of MI-219 with oxaliplatin resulted in 767 genes being altered that were not significantly related to MI-219-oxaliplatin synergy. The most intriguing aspect of this finding is the emergence of 286 synergy-specific unique genes that were not found in MI-219 or oxaliplatin single treatment group (Fig 2 A). These findings confirm that synergy between MI-219 and oxaliplatin emanates from a well orchestrated set of specific genetic perturbations. This is further supported by our ingenuity analysis of combination and synergy unique gene pools that showed some highly contrasting differences between combination events and synergistic events. The synergy unique 287 genes were pooled into 14 closely knit functional groups as demonstrated through the ingenuity pathway system. Significantly, in contrast to the enrichment of large set of canonical pathways in total combinations genes, the specific synergy-unique associated 14 networks were restricted to cancer, gastrointestinal disease, genetic disorder, cell cycle and cell death pathway.

EGR1 is well recognized to directly induce the transcription of p53 and binds to p53 forming a cytoplasmic complex [31]. Direct binding of the p53 promoter by Egr1 was first shown in human melanoma A375-C6 cells during thapsigargin-induced apoptosis [32]. Studies of the Egr1-null mouse system have revealed that Egr1 normally controls p53 expression leading to cell cycle arrest and senescence [33]. These cells express little or no p53 although the gene is intact. This suggested that p53 activation by MDM2 inhibitor as single agent or in combination with oxaliplatin could be aided by EGR1 co-expression. In line with this, we observed activation of EGR1 network by MDM2-oxaliplatin treatment and this was validated at the protein level in PDAC, colon and breast cells. Furthermore, siRNA silencing of EGR1 abrogated MI219-oxaliplatin efficacy and thus, reaffirmed the critical role of EGR1 in synergy of the two drugs.

CREB binding protein (CREBBP) is a large (~300 KDa) pleiotropic cellular coactivator protein critical to the execution of virtually all known cellular programs, including cell growth, differentiation, the integration of both signal-dependent and -independent cellular responses, and apoptosis. CREBBP and its sister protein p300 are highly conserved in multicellular organisms and have been shown to have profound effects on somatic differentiation during early embryogenesis [34,35]. The transcriptional activity of p53, which is tightly linked to its tumor suppressor function, appears to depend upon efficient recruitment of CREBBP to p53 target promoters. Consistent with this observation, recent studies have shown that the activation domain of p53 participates in CREBBP recruitment [36]. Taken together, these findings indicate that CREBBP plays a critical role in supporting p53-dependent transcription function. Results from our network modeling also demonstrated a statistical enhancement of CREBBP hub and western blotting verified this activation at the protein level. Similar to EGR1, siRNA silencing of CREBBP resulted in diminished MI-219-oxaliplatin activity in PDAC, colon and breast cells. These results again indicate that CREBBP plays a definite role in optimizing p53 re-activation by MI219-oxaliplatin.

MI-219-oxaliplatin network analysis also showed emergence of a driver NF-κB network. NF-κB is a master transcriptional regulator that is known to regulate a number of critical genes governing normal homeostasis of cell [37]. In cancer, activation of NF-κB has been associated with poor prognosis and resistance to drug treatment [38]. MI-219-oxaliplatin drug response signatures showed that NFκB expression was downregulated and secondary networks (EGR1 hub) were activated that drove growth inhibition and apoptosis. We also observed constitutive activation of the NF-κB-CREBB axis. This points to the previously unrecognized role of NF-κB in p53 mediated apoptosis. However, this regulatory network especially in response to MI-219, oxaliplatin or combination is still poorly understood and requires further investigation.

Pharmaceutical strategies to re-activate p53 has been a topic of intense research in recent years and a number of potent small molecules are either in PhaseI/II or entering clinical trial [13]. Instead of rushing these promising MDM2 inhibitors into the clinic, a comprehensive understanding of their mechanism of action is first, absolutely necessary and network modeling can aid in this effort. In conclusion, we have demonstrated by using network modeling, that unique driver networks which play a critical role in MI-219-oxaliplatin synergy can be identified and functionally verified. These synergy networks coerce an orchestrated set of events that cumulatively optimize p53 re-activation resulting in enhanced growth inhibitory and apoptotic effects in different wt-p53 cell lines. The results provide strong rationale for the design of novel treatment strategies using this highly potent combination in patients carrying tumors exhibiting wt-p53.

## MATERIAL AND METHODS

### Cell culture, experimental reagents and chemicals

Capan-2 (PDAC; wt-p53) cells were purchased from American Type Culture Collection (ATCC). HCT-116 (Colon cancer; wt-p53) cells were generously provided by Dr. Vogelstein's group. MCF-7 (Breast cancer; wt-p53) cells were obtained Karmanos Cancer Institute, Detroit Michigan. These cell lines have been tested and authenticated in our core facility, Applied Genomics Technology Center at Wayne State University, as late as March 13, 2009. The method used for testing was short tandem repeat (STR) profiling using the PowerPlex® 16 System from Promega (Madison, WI). Primary antibodies for p53, EGR1, CBP300 (a CREBBP), E Cadherin, MDM2 and p21 were purchased from Cell Signaling (Beverly MA). All secondary antibodies were obtained from Sigma (St. Louis, MO). MI-219 was synthesized by using our previously published methods [39,40].

### Cell growth inhibition studies by MTT and clonogenic assay

Capan-2, HCT-116 and MCF-7 cells were seeded in a 96-well culture plate (at a density of 3 x 10^3^ cells per well) and treated with different ratios of MI-219 (0-30 μM), or oxaliplatin (0-30 μM) or their combination for 32 hrs and MTT assay was performed as described earlier [41]. The results were plotted as means ± SD of three separate experiments using six determinations per experiment for each experimental condition. Clonogenic assay for cell survival on the three cell lines was performed according to previously described methods [42].

### Quantification of apoptosis by Annexin V FITC flow cytometry and ELISA

Apoptosis in Capan-2, HCT-116 and MCF-7 cells was determined using Annexin V FITC apoptosis kit (Biovision Research Products) and ELISA detection kit (Roche, Palo Alto, CA) according to manufacturer's protocol and published previously [43].

### siRNA and transfections

To study the effect of MI-219-oxaliplatin combination on re-activating p53 in the presence of p53 siRNA, E-Cadherin siRNA, EGR1 siRNA and p65 siRNA and CDKN2A (p16) siRNA, we utilized siRNA silencing technology in Capan-2, HCT-116 and MCF-7 cells. The respective siRNAs and control siRNA were obtained from Cell Signaling. Cells were transfected with either p53 siRNA, E-Cadherin siRNA, EGR1 siRNA or p65 siRNA for 5 hrs using LipofectAMINE 2000 according to the manufacturer's protocol (Cell Signaling). After the siRNA treatment period cells were further treated with MI-219-oxaliplatin combination in 96 well plates for MTT and 6 well plates for Annexin V FITC assays respectively.

### Western blot analysis

Capan-2, HCT-116 and MCF-7 cells were exposed to either MI-219, oxaliplatin or their combination for 16 and 32 hrs followed by extraction of protein for western blot analysis. Preparation of cellular lysates, protein concentration determination and SDS-PAGE analysis has been previously described. [44].

### Microarrays and Gene Expression Analysis

Gene expression networks from Capan-2 cells were determined from an analysis of global gene expression time series data. Cells were plated so that they reached 75% confluence after 3 days. At this point cultures were treated with either MI-219 (15 μM) or oxaliplatin (15 μM) or their combination and RNA was isolated from triplicate parallel plated plates at 16 and 32 hours after addition of inhibitor. Media was changed the day after plating and at the start of treatment. Quantitative measurement and the high quality of all mRNA samples were assured by analysis with the NanoDrop 1000, Agilent Bioanylizer and the Agilent RNA 6000 Nano Kit (Agilent Technologies, Waldbronn, Germany). Expression levels at each time point for each cell and treatment were determined by microarray analyses using the Illumina human HT12 array. Data were processed for quality control and normalized across compared arrays by quantile normalization. Genes with 1.7 or greater expression fold-change at any time point in the series were included in Ingenuity Pathways Analyses. Cluster analysis of expression profiles was performed with Bayesian analysis using CAGED software. Canonical pathways analysis identified the pathways from the Ingenuity Pathways Analysis library of canonical pathways that were most significant to the data set. Molecules from the data set that met the 1.7 fold-change cut-off and were associated with a canonical pathway in Ingenuity's Knowledge Base were considered for the analysis. The significance of the association between the data set and the canonical pathway was measured in 2 ways: 1) A ratio of the number of molecules from the dataset that map to the pathway divided by the total number of molecules that map to the canonical pathway. 2) Fisher's exact test was used to calculate a p-value determining the probability that the association between the genes in the dataset and the canonical pathway is explained by chance alone.

## 






